# Blood Cancer and the Heart: Light Chain Cardiomyopathy in Refractory Multiple Myeloma

**DOI:** 10.1155/2022/7846846

**Published:** 2022-07-30

**Authors:** Abdulbaril Olagunju, Chandana Shekar, Michael Morris, Anantharam Kalya, Farouk Mookadam, Samuel Unzek

**Affiliations:** ^1^Department of Medicine, Creighton University, Phoenix, AZ, USA; ^2^Heart Center, Banner University Medical Center, University of Arizona College of Medicine-Phoenix, Phoenix, AZ, USA; ^3^Department of Diagnostic Radiology, Banner University Medical Center, University of Arizona College of Medicine-Phoenix, Phoenix, AZ, USA

## Abstract

We report a case of a 57-year-old woman with a history of multiple myeloma (MM) and light chain (AL) amyloidosis who presented due to worsening dyspnea on exertion. Her MM has been refractory to multiple chemotherapy regimens and two autologous bone marrow transplantation. Diagnostic evaluations including serum kappa and lambda chains, echocardiogram, pyrophosphate cardiac scan, and cardiac magnetic resonance were indicative of a progression to AL cardiomyopathy. Addition of daratumumab to her regimen appeared to ameliorate the progression of AL cardiomyopathy. However, it was stopped due to adverse effects of pancytopenia and allergic reactions including skin rash and hives. She was hospitalized for heart failure exacerbation and died approximately 2 months following the discontinuation of daratumumab. This case highlights the late presentation of AL cardiomyopathy in refractory MM.

## 1. Introduction

Multiple myeloma (MM) is a plasma cell malignancy that is responsible for approximately 10% of all hematologic malignancies [1, 2]. As of 2015, about 124,733 were living with MM in the United States; an additional 32,000 new cases were diagnosed in the year 2020, accounting for 1.8% of new cancer diagnosis [1]. Approximately 12-15% of MM patients develop light chain (AL) amyloidosis [2]. In these patients, the progression to AL cardiomyopathy remains a significant cause of morbidity and mortality [3]. Here, we present a case of refractory MM that was complicated by AL cardiomyopathy.

## 2. Case Presentation

A 57-year-old woman with a history of refractory MM and AL amyloidosis presented to the emergency department with acute exertional dyspnea and palpitations. She denied cough, chest pain, paroxysmal nocturnal dyspnea, fever, chills, and smoking history. Her MM was refractory to multiple combination of treatments including lenalidomide/bortezomib/dexamethasone, carfilzomib/pomalidomide/dexamethasone, high-dose melphalan with autologous bone marrow transplant, pomalidomide/ixazomib/prednisone, cyclophosphamide/bortezomib/dexamethasone (CyBorD), isatuximab/pomalidomide, and a second autologous bone marrow transplant with carfilzomib/lenalidomide/dexamethasone.

Physical exam was notable for jugular venous distention and bilateral lower extremity pitting edema. There were no murmurs, rubs, or gallops. The differential diagnoses included acute systolic heart failure due to cancer-related drug treatments, atrial fibrillation with rapid ventricular response (AFRVR) with acute diastolic heart failure, acute myocardial infarction, and acute pulmonary embolism.

**Figure 1:**
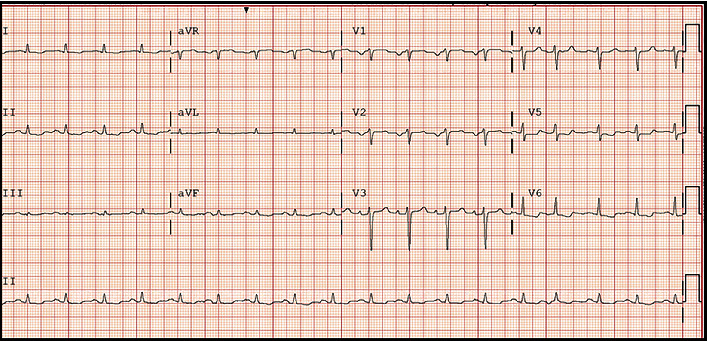
ECG showed sinus rhythm with low voltage in the limb leads.

**Figure 2:**
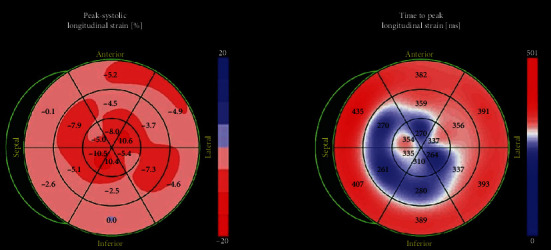
Echocardiographic polar map highlighted a decrease in global longitudinal strain.

**Figure 3:**
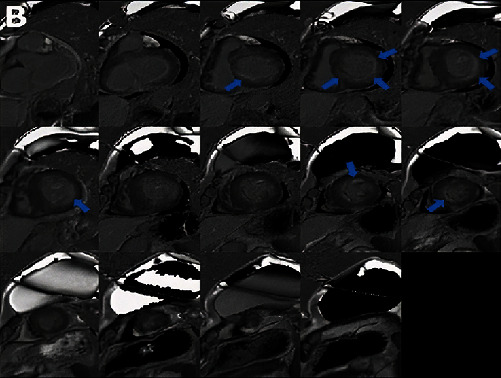
CMR revealed patchy midmyocardial and subendocardial LGE.

**Figure 4:**
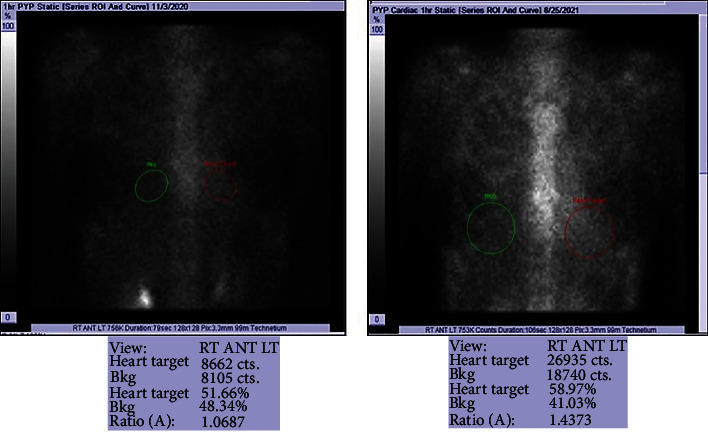
Right PYP scan showed increased PYP uptake compared to the left PYP scan done almost a year earlier.

Vital signs were remarkable for heart rate of 101 beats per minute (bpm). Laboratory data were remarkable for hemoglobin 63 g/L (normal range, 135-170 g/L), platelets 54 K/*μ*L (normal range, 130-450 K/microL), white blood cell 3.9 × 10^9^/L (normal range, 4.0-11.0 × 10^9^/L), NT-proBNP 4,375 pmol/L (normal range, <14.8 pmol/L), troponin 117 mcg/L (normal range, <14 mcg/L), and serum kappa and lambda chains 1.62 mg/L and 1302 mg/L (normal range, 3.3 to 19.4 mg/L and 5.71 to 26.3 mg/L, respectively). Twelve lead electrocardiogram (ECG) showed sinus tachycardia with low voltage in the limb leads (Figure 1). Computed tomography angiogram of the chest was negative for pulmonary embolism. Echocardiogram revealed left ventricular (LV) ejection fraction of 65%, severely increased LV posterior wall end diastolic thickness (17 mm) (normal: 6 to 9 mm), grade II diastolic dysfunction, mildly enlarged right ventricular (RV) chamber with mildly reduced systolic function, RV systolic pressure of 56 mmHg, and reduced peak average global longitudinal strain with apical sparing of the LV on strain imaging and polar map (Figure 2). Cardiac magnetic resonance (CMR) showed thick myocardial walls with patchy subendocardial late gadolinium enhancement (LGE) prominent along the lateral, inferoseptal walls from the base through the midventricle and papillary muscles (Figure 3). T1 times were very elevated compared to CMR from a year earlier suggesting progressive cardiomyopathy. Basal anteroseptal and inferolateral wall thickness increased from 17-22 mm and 13-19 mm, respectively. No focal regional wall motion abnormality was identified. A pyrophosphate (PYP) cardiac scan compared to a year earlier was positive for scintigraphic evidence of evolving amyloid deposition (Figure 4).

She was transfused with red blood cells with a target hemoglobin of at least 8 g/dL. Diuresis was initiated with intravenous furosemide. This significantly improved her dyspnea and reduced ankle swelling. She was transitioned to torsemide at discharge. Daratumumab (Darzalex) was added to her MM regimen: daratumumab/melflufen/dexamethasone. Due to the refractoriness of her MM, she was not a candidate for heart transplant. At 6-month follow-up, she was asymptomatic, and her cardiac and renal function remained stable. However, a bone marrow biopsy showed stable clonal plasma cells (13%). Furthermore, daratumumab was stopped due to worsening pancytopenia and allergic reactions including skin rash and hives. Her cardiac and renal function remained stable; however, she complained of progressive neuropathy. She was readmitted approximately two months following discontinuation of daratumumab for an acute exacerbation of her heart failure. She suddenly became tachycardic with a heart rate of 140 bpm and was unresponsive and noted to have pulseless electrical activity (PEA). A cardiopulmonary resuscitation was not performed due to her wishes. The cause of her death was thought to be an obstructive shock due to a massive pulmonary embolism.

## 3. Discussion

Approximately 50% of patients with AL amyloidosis have cardiac involvement [3]. The development of heart failure symptoms portends a poor prognosis with a median survival < 6 months in patients without adequate management of the instigating plasma cell dyscrasia [3].

Echocardiogram in the early stage typically reveals a preserved ejection fraction; however, a reduction in the global longitudinal strain that spares the cardiac apex and manifests in a reduction in stroke volume index indicates significant cardiac dysfunction [4, 5]. In addition, a symmetric biventricular increased wall thickness may be noted on echocardiogram, creating abroad differential for thick-walled cardiomyopathies [4]. CMR is important in the diagnosis of cardiac amyloidosis to assess extent and severity, with tissue biopsy being the gold standard of course [4, 5]. LGE is a significant prognostic factor that correlates with the degree of cardiac infiltration by the light chains [5], with subendocardial LGE in early stages of the disease and transmural LGE seen in in the later stages of the disease [5]. Furthermore, CMR T1 relaxation time is directly proportional to the degree of cardiac light chain infiltration and hence serves a useful method to quantify the progression of cardiac involvement [5]. Unlike transthyretin amyloidosis, PYP scan is positive only in about 40% of AL cardiomyopathy cases [6]. In patients with unconfirmed AL amyloidosis, a tissue biopsy which typically reveals an apple-green birefringence on polarized light microscopy is required [7]. This should be followed by further evaluation to assess for the presence of MM through bone marrow plasma cell quantification, quantification of AL in serum and urine, lytic lesions in skeleton, presence of soft tissue deposits, and other end organ manifestation as described by the International Myeloma Working Group 2014 criteria [8].

The management of heart failure symptoms in patients with AL cardiomyopathy is different from other forms of heart failure [9]. Diuresis with loop diuretics and aldosterone receptor antagonists are important in achieving and maintaining euvolemia [9]. Except in patients with AFRVR, beta-blockers (BB) are generally avoided because they worsen hypotension by suppressing the compensatory sinus tachycardia required to maintain normotension [9]. Angiotensin-converting enzyme inhibitors and angiotensin receptor blockers are also not well tolerated because they aggravate hypotension [9]. Digoxin binds to the amyloid fibrils which increases the risk for digoxin toxicity [9]. Hence, it must be used with caution in patients with AFRVR that cannot tolerate BB due to hypotension [9]. Calcium channel blockers are typically avoided because their negative inotropic effect worsens heart failure symptoms [9].

In addition to relieving heart failure congestion with diuresis, the prognosis of AL cardiomyopathy centers on the treatment of the underlying plasma cell dyscrasia [3]. The available treatment options include hematopoietic stem cell transplantation (HCT) and/or chemotherapy [3]. The combination of high dose melphalan and HCT is associated with a high rate of complete hematologic and organ response and overall survival [3]. However, the significant treatment-related mortality in patients with AL amyloidosis makes the combination available to only 25-30% of patients [3]. These are patients without advanced cardiomyopathy, advanced age, multiple organ involvement, hypotension, and low serum albumin levels [8]. The prognosis of AL amyloidosis can be assessed using the European modification of the Mayo 2004 staging [10]. The staging is based on the serum concentration of the NT-proBNP and troponin; the cut-off values are 332 ng/L (normal range, ≤169.4 ng/L) and 0.035 mcg/L (normal range, ≤0.1 mcg/L), respectively [9] (Table 1).

Traditionally, patients who are ineligible for HCT have been treated either with melphalan and dexamethasone or bortezomib-based regimen including bortezomib, melphalan, and dexamethasone or CyBorD [11]. Bortezomib should be used with caution due to its association with worsening heart failure [12]. CyBorD is a more commonly used regimen based on outcomes of trials that demonstrated higher hematologic and organ response compared to BMD [11]. Daratumumab, a monoclonal antibody against the CD38 protein expressed on the clonal plasma cells, was associated with a shorter time to first hematologic and deep response as well as a highly significant improvement in cardiac and renal response in the ANDROMEDA trial either alone or in combination with CyBorD [10–12]. These outcomes were also the highlight of phase II trials that evaluated the impact of daratumumab in patients with refractory and relapsed AL amyloidosis [12]. CD38 plays a role in protecting myeloma cells from immune system destruction [11]. Hence, the level of CD38 expression in AL amyloidosis is proportional to the severity of cardiac involvement and the serum concentration of NT-proBNP [12]. The binding of daratumumab to CD38 results in immune-mediated plasma cell death via multiple mechanisms including apoptosis, complement-dependent cytotoxicity, antibody-dependent cellular cytotoxicity, and antibody-dependent cellular phagocytosis [11]. Besides daratumumab, doxycycline has been associated with a higher rate of organ response and survival in a retrospective study [13]. However, in a recent randomized controlled trial, doxycycline did not prolong survival [14].

Our patient had Mayo stage IIIB AL amyloidosis that remained refractory to multiple chemotherapy regimens and two HCTs, and hence, daratumumab was added with the hope of achieving some cardiac response. It is likely that her readmission for acute exacerbation of heart failure was associated with the discontinuation of daratumumab.

## 4. Conclusion

We present a case of refractory MM complicated by progressive AL cardiomyopathy in the setting of AL amyloidosis. In general, the prognosis is poor. Daratumumab is a novel immune modulating agent that may help even after a few doses and offers hope for cardiac response. However, our patient discontinued it after 6 months due to worsening pancytopenia and allergic reactions. This was followed by hospitalization for acute heart failure exacerbation during which she developed a PEA and expired.

## Figures and Tables

**Table 1:** Cut-off values for NT-proBNP and troponin based on the European modification of the Mayo 2004 staging for prognosticating AL amyloidosis.

Stage	Markers
I	Troponin < 0.035 mcg/L & NT‐proBNP < 332 ng/L
II	Troponin ≥ 0.035 mcg/L or NT‐proBNP ≥ 332 ng/L
IIIa	Troponin ≥ 0.035 mcg/L & NT‐proBNP < 8500 ng/L
IIIb	Troponin ≥ 0.035 mcg/L & NT‐proBNP ≥ 8500 ng/L
